# Maximizing QRS duration reduction in contemporary cardiac resynchronization therapy is feasible and shorter QRS duration is associated with better clinical outcome

**DOI:** 10.1007/s10840-022-01463-y

**Published:** 2023-01-11

**Authors:** Rasmus Borgquist, Sofia Marinko, Pyotr G Platonov, Lingwei Wang, Uzma Chaudhry, Johan Brandt, David Mörtsell

**Affiliations:** 1 Cardiology section, Department of clinical sciences, Lund University, Lund, Sweden; 2 Arrhythmia section, Skane University Hospital, Entrégatan 7, 222 42 Lund, Sweden

**Keywords:** CRT, Heart failure, Device optimization, Mortality, Heart failure hospitalization

## Abstract

**Background:**

We aimed to evaluate if optimization by maximizing QRS duration (QRSd) reduction is feasible in an all-comer cardiac resynchronization therapy (CRT) population, and if reduced, QRSd is associated with a better clinical outcome.

**Methods:**

Patients with LBBB receiving CRT implants during the period 2015–2020 were retrospectively evaluated. Implants from 2015–2017 were designated as controls. Starting from 2018, an active 12-lead electrogram-based optimization of QRSd reduction was implemented (intervention group). QRSd reduction was evaluated in a structured way at various device AV and VV settings, aiming to maximize the reduction. The primary endpoint was a composite of heart failure hospitalization or death from any cause.

**Results:**

A total of 254 patients were followed for up to 6 years (median 2.9 [1.8–4.1]), during which 82 patients (32%) reached the primary endpoint; 53 deaths (21%) and 58 (23%) heart failure hospitalizations. Median QRS duration pre-implant was 162 ms [150–174] and post-implant 146ms [132–160]. Mean reduction in QRS duration was progressively larger for each year during the intervention period, ranging from − 9.5ms in the control group to − 24 in the year 2020 (*p* = 0.005). QRS reduction > 14 ms (median value) was associated with a lower risk of death or heart failure hospitalization (adjusted HR 0.54 [0.29–0.98] (*p* = 0.04).

**Conclusions:**

Implementing a general strategy of CRT device optimization by aiming for shorter QRS duration is feasible in a structured clinical setting and results in larger reductions in QRS duration post-implant. In patients with a larger QRS reduction, compared to those with a smaller QRS reduction, there is an association with a better clinical outcome.

**Graphical Abstract:**

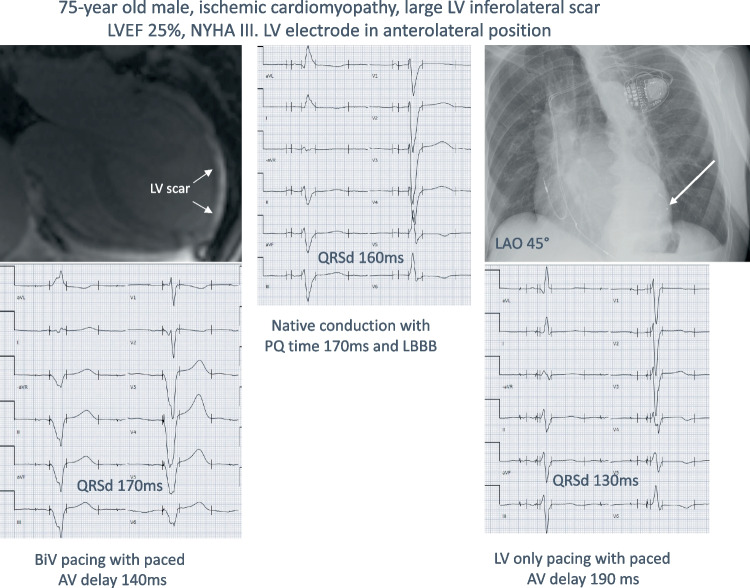

## Background

Introduction: Cardiac resynchronization therapy (CRT) is an established treatment for heart failure in selected patients [1]. The primary aim of CRT is to resynchronize a dyssynchronous contraction of the left ventricle. Dyssynchrony may be caused by left bundle branch block or other conduction disturbances, and current guidelines emphasize the importance of prolonged QRS duration (QRSd) in the selection criteria for suitable candidates, where a class I indication is given only to patients with LBBB and QRS duration ≥ 150 ms, and patients with non-LBBB have a stronger recommendation if the QRSd is ≥ 150 ms (IIa) compared to < 150ms (IIb) [2]. Similarly, the magnitude of reduction of QRS duration has in several studies been associated with better clinical outcome and higher probability of echocardiographic reverse remodeling [3]. However, there are still a significant number of patients in this group who do not improve after CRT.

It is well-known that individual programming of the device’s timing of atrioventricular (AV) and ventriculo-ventricular (VV) delay can be important in order to maximize the benefit of CRT [4]. The introduction of quadripolar electrodes and device-based algorithms for optimization of AV and VV delays and delivery of LV-only pacing and fusion pacing have greatly increased the programming options in each individual CRT-treated patient. All major vendors of CRT devices now have built-in optimization algorithms in their devices, and most of these algorithms have shown non-inferiority to echocardiography-optimized device settings regarding short-term outcome. Head-to-head comparisons between different vendors’ algorithms have not been performed, and it is not clear which is the best strategy for optimizing device settings. It would be appealing to apply a uniform (validated) optimization strategy to all patients, regardless of device brand. Retrospective studies have consistently indicated that a larger reduction in QRS duration is associated with better outcome, as well as improved reverse remodeling [3]. A recent study has also shown that by combining the built-in algorithm (in this case the Sync AV algorithm) with individually tailored AV delay, it was possible to obtain a greater mean reduction in QRSd, compared to using the algorithm alone [5].

We aimed to evaluate if it is feasible to obtain additional QRS reduction, on top of the built-in algorithms in the device, by adjusting AV and VV delays in a structured way, in an all-comer CRT population. We also aimed to assess whether larger QRS reduction was associated with better clinical outcome.

We aimed to develop and implement a pragmatic vendor-independent strategy for CRT optimization in a tertiary care referral center, and to evaluate if the successful implementation of this optimization scheme resulted in better clinical outcome.

## Methods

The study was performed in a tertiary care center. Medical records of 254 consecutive patients with left bundle branch block (LBBB) according to the 2018 ACC/AHA/HRS criteria and a class I indication for CRT, during the period 2015-2020, were retrospectively evaluated [6]. The right atrial lead was typically placed in the right appendage, the right ventricular lead in the apex or septum (operator’s preference). The coronary sinus lead was placed in a lateral, posterolateral or posterior position if possible, and anterior position only as a last resort. Left ventricular lead position was retrospectively evaluated in the left anterior oblique and right anterior oblique views by an experienced electrophysiologist (RB), using the 17-segment model, and positions were split into lateral (anterolateral or inferolateral), anterior, inferior, or apical position [7].

**Fig. 1:**
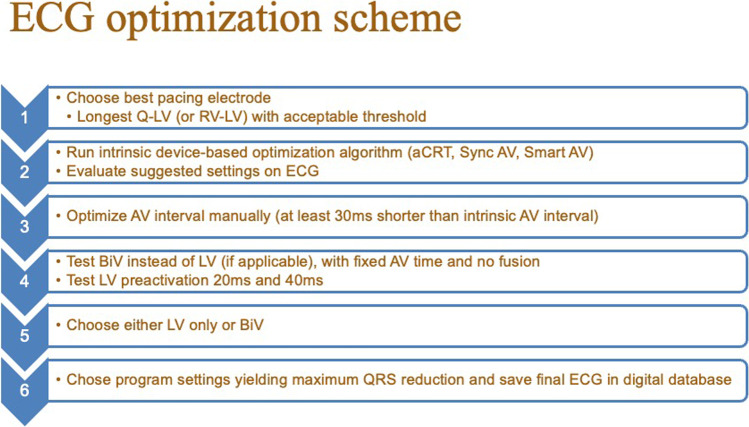
ECG optimization scheme

Implants performed during the first 3 years (2015-2017) were designated as the control group, and in these patients, the suggested settings from the device-based algorithms were used, if applicable. This included primarily the aCRT algorithm from Medtronic and the QuickOpt algorithm from Abbott [8, 9]. For patients where the algorithms could not be used, typical programming in the control group included a fixed AV time at least 20 ms shorter than the intrinsic conduction time to ensure biventricular capture and simultaneous pacing of the right and left ventricle, or (in the case of permanent atrial fibrillation) synchronous biventricular pacing without trigger mode. Starting in 2018, an active 12-lead electrocardiogram (ECG)-based optimization of QRS duration reduction post-implant was implemented, and these patients were designated as the intervention group. There was a gradual implementation, and the strategy was fully implemented in 2020 and onwards, where all patients routinely went through the optimization process. The method is summarized in Fig. 1. Starting in the year 2018, postoperative QRS duration and morphology were evaluated in a structured stepwise way at various device settings, including the use of specific device algorithms when applicable (AdaptiveCRT, SyncAV, SmartDelay) with manual modifications of AV and VV delays and LV-only pacing when applicable, aiming to maximize the reduction of the QRS duration. A pacing electrode was chosen based on the longest Q-LV or longest RV-LV conduction time (if no intrinsic AV conduction). If the best vector had a threshold at or above the limit of 3.0 V/1.0 ms, then the second-best vector was chosen. If two vectors were similar, the one with the lowest threshold and/or highest impedance was chosen. Vectors with diaphragmatic stimulation were not considered suitable and hence excluded. The suggestion from the device-based algorithm was then tested and evaluated using 12-lead ECG with different AV intervals (as suggested by Varma et al. [5]). If LV-only or fusion pacing was the suggested setting, then a standard BiV setting was also tested. For the BiV setting, a fixed AV delay at least 20 ms shorter than intrinsic conduction was chosen, typically 140/170 ms, or shorter if needed. Finally, LV pre-activation was evaluated with – 20 ms and – 40 ms respectively. The setting with the overall narrowest QRS complex was then chosen. If two settings were similar in QRS duration, a morphology with visible LV pre-activation (i.e., early positive deflection in lead V1 and/or lead I) was favored. Optimization was performed immediately post-operatively or the day after. If the chosen settings resulted in subjective improvement and no objective signs of worsened heart failure, the settings were kept the same at follow-up visits. Follow-up interval was typically at 2 months, then at 6 months (for non-responders) and then every 12 months, plus continuous remote monitoring. Digital ECGs before and after CRT implantation were collected and QRS duration reduction was automatically analyzed, with manual inspection and validation of correct position of the automatic timing calipers.

The primary endpoint was a composite of hospitalization due to heart failure or death from any cause.

### Statistical methods

SPSS version 27 (IBM) was used for statistical analyses. Normally distributed data is presented as mean ± standard deviation; non-normally distributed data is presented as median [interquartile range]. Cox regression analysis was used in time-dependent analysis to evaluate the hazard ratio for the primary composite endpoint (death and heart failure hospitalization). Variables with a univariable *p*-value < 0.10 were entered into a multivariable model. A Kaplan-Meier analysis with log-rank test was used to compare survival between the different time periods, and for groups with different magnitudes of QRSd reduction. For the comparison between the two implant periods, the time of follow-up was capped at 2 years, to eliminate the different times of follow-up inherent to the study design. For all analyses, a two-sided *p*-value < 0.05 was considered significant.

## Results

A total of 254 patients were included and were followed for up to 6 years (median 2.9 [1.8–4.1] years). The time spent on postoperative optimization was not uniformly recorded, but typically varied between 15 and 45 min.

**Fig. 2:**
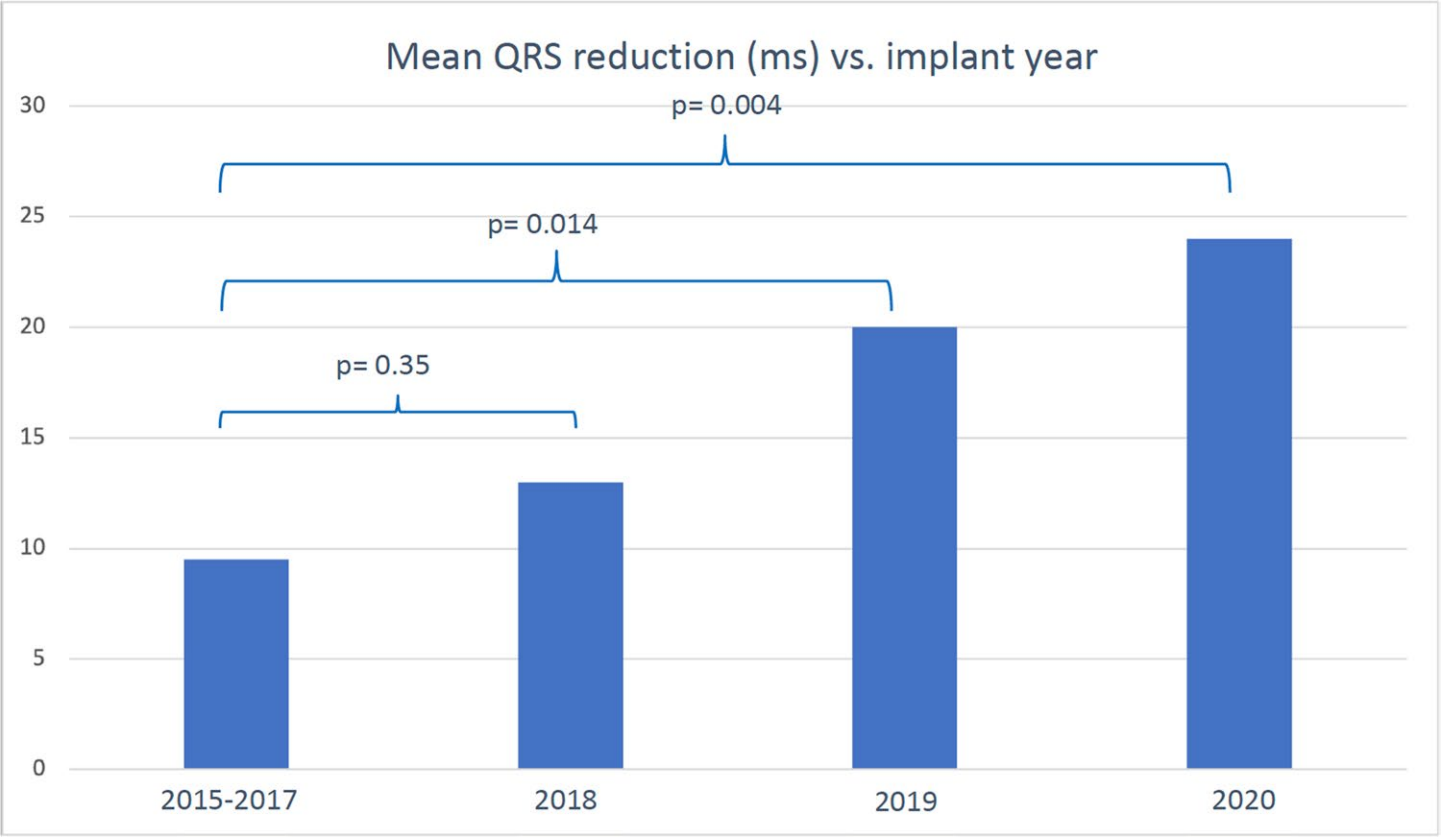
QRS duration reduction per implant period (years)

During follow-up, 82 patients (32%) reached the primary endpoint; in total, there were 53 deaths (21%) and 58 (23%) heart failure hospitalizations. Baseline demographic data is presented in Table 1. Median QRS duration pre-implant during the entire time period was 162 ms [150–174] and post-implant 146 ms [132–160]. Progressively, more patients underwent structural QRSd reduction evaluation for each year, and correspondingly, the mean reduction in QRS duration was progressively larger for each year during the intervention period, changing from − 9.5ms in the control group to − 24 in the year 2020 (*p* = 0.005) (Fig. 2). The use of LV-only pacing algorithms, AV and VV times are reported in Table 1. LV-only pacing was used more often during the intervention period, compared to the control period, but the reduction in QRS duration was not significantly different between those with LV-only pacing and patients with biventricular pacing from both RV and LV electrodes. Overall, at the group level, the sensed and paced AV times did not differ significantly between the control and intervention period, but LV pre-activation was shorter in the intervention period.

**Table 1:** Baseline characteristics stratified for implant period

	2015-2017	2018-2020	*p*-value
	*N* = 116	*N* = 138	
Age (years)	70 ± 9.9	71 ± 10	0.42
Hypertension (%)	81 (70%)	91 (66%)	0.59
CABG (%)	18 (15%)	27 (20%)	0.41
Diabetes (%)	46 (40%)	39 (29%)	0.06
Atrial fibrillation (%)	48 (42%)	54 (40%)	0.72
Paroxysmal (%)	24 (21%)	31 (23%)	0.88
Chronic (%)	24 (21%)	23 (17%)	0.51
Female (%)	22 (19%)	34 (25%)	0.29
CRT-D (vs CRT-P) (%)	87 (75%)	90 (65%)	0.10
Ischemic etiology (%)	49 (42%)	56 (41%)	0.80
LVEF (%)	25 ± 6.9	27 ± 5.6	0.04
Hemoglobin	136 ± 19	132 ± 15	0.07
NT-ProBNP (per 100 units)	1857 [886–4931]	1212 [487–2672]	0.008
ACE inhibitor/ARB/ARNi (%)	108 (93%)	129 (94%)	0.91
Betablocker (%)	101 (87%)	116 (84%)	0.59
Aldosterone antagonist (%)	70 (60%)	76 (55%)	0.45
Digoxin (%)	10 (9%)	7 (5%)	0.32
Loop diuretics (%)	86 (74%)	77 (56%)	0.003
NYHA class (I/II/III/IV)	2/36/54/8	4/40/50/6	0.51
Device brand			0.001
Medtronic	26 (22%)	41 (30%)	
Abbott	88 (76%)	75 (54%)	
Boston	2 (2%)	21 (15%)	
Biotronik	0	1 (1%)	
QRS duration pre-CRT (ms)	162 ± 18	161 ± 14	0.37
QRS duration post-CRT (ms)	153 ± 23	143 ± 20	<0.001
QRS reduction (ms)	9.5 ± 24	18 ± 23	0.004
Left ventricular lead position	*N* = 200	*N* = 315	
Lateral	136 (68%)	227 (72%)	0.32
Anterior	39 (15%)	22 (7%)	0.04
Inferior	7 (4%)	19 (6%)	0.22
Apical	27 (14%)	47 (15%)	0.70
AV node ablation	4 (3.4%)	5.8% (8)	0.28
LV-only pacing algorithm activated	9 (8%)	29 (21%)	0.002
Sensed AV time (ms)	110 [90–150]	110 [90–220]	0.11
Paced AV time (ms)	150 [130–200]	160 [130–250]	0.12
VV time (LV preactivated, ms)	25 [0–65]	0 [0–50]	0.002
Follow-up time (years)	4.1 [3.4–4.9]	1.9 [1.6–2.6]	< 0.001

*CABG* coronary artery bypass graft, *LVEF* left ventricular ejection fraction, *ACE* angiotensin-converting enzyme, *ARB* angiotensin receptor type II blocker, *ARNi* angiotensin receptor blocker neprilysin inhibitor, *NYHA* New York heart association classification of heart failure. Mean ± standard deviation is reported for normally distributed variables; otherwise, median [interquartile range] is reported

During the intervention period, fewer patients had their LV leads placed in an anterior position. Cox regression analysis was used to determine the hazard ratio for QRS duration reduction with regards to the combined primary endpoint; HR 0.89 [CI 0.80–0.99] per 10-ms QRS reduction, *p* = 0.037. If QRS duration reduction was dichotomized using the median value (− 14 ms), the corresponding HR for QRS reduction ≥ 14 ms was 0.57 [0.33–0.98] *p* = 0.038, compared to QRS reduction < 14 ms (Table 2). In a multivariate Cox regression model, variables with *p* < 0.05 were entered. The final model was thus adjusted for age, gender, NYHA class, ischemic etiology, CRT-P/CRT-D, left ventricular ejection fraction (LVEF), and diabetes, and the adjusted hazard ratio for larger QRS reduction was 0.54 [0.29–0.98] (*p* = 0.04).

**Table 2:** Cox regression analysis for risk of death or hospitalization for heart failure within 2 years post-implant

	Cox regression			
	Univariate		Multivariate	
	Hazard ratio(95% CI)	*p*-value	Hazard ratio(95% CI)	*p*-value
Age(years)	1.03 (1.00–1.05)	**0.03**	0.99 (0.95–1.03)	0.45
Hypertension(%)	1.13 (0.63–2.00)	0.67		
CABG(%)	0.79 (0.37–1.67)	0.54		
Diabetes(%)	1.05 (0.60–1.83)	0.87		
Atrial fibrillation(%)		0.058		0.82
Paroxysmal(%)	1.37 (0.71–2.67)	0.35	0.83 (0.39–1.78)	0.64
Chronic(%)	2.13 (1.14–3.98)	**0.017**	1.11 (0.51–2.41)	0.80
Female(%)	1.70 (0.80–3.60)	0.17		
CRT-D (vs CRT-P)(%)	1.47 (0.85–2.43)	0.17		
Ischemic etiology(%)	1.21 (0.71–2.05)	0.49		
LVEF(%)	0.95 (0.91–0.98)	**0.006**	0.98 (0.93–1.03)	0.42
Hemoglobin	0.97 (0.96–0.99)	**0.001**	0.98 (0.97–1.00)	0.05
NT-ProBNP (per 100 units)	1.01 (1.01–1.02)	**< 0.001**	1.01 (1.00–1.01)	**< 0.001**
ACE inhibitor(%)	1.34 (0.65–2.73)	0.43		
Betablocker(%)	1.19 (0.54–2.63)	0.67		
Aldosterone antagonist(%)	0.98 (0.57–1.67)	0.93		
Digoxin(%)	0.78 (0.25–2.50)	0.68		
Loop diuretics(%)	3.65 (1.73–7.73)	**0.001**	2.38 (1.03–5.48)	**0.04**
NYHA class(I/II/III/IV)	2.67 (1.73–4.10)	**< 0.001**	1.93 (1.12–3.33)	**0.018**
Anterior LV lead location	0.91 [0.39–2.1]	0.83		
LV-only pacing algorithm activated	0.66 [0.30–1.5]	0.30		
QRS duration pre-CRT(ms)	0.89 (0.75–1.05)	0.15		
QRS duration post-CRT(ms)	1.08 (0.96–1.21)	0.22		
QRS reduction** > **14 ms	0.57 (0.33–0.98)	**0.04**	0.42 (0.22–0.80)	**0.008**

Figures in bold represent statistically significant associations (*p *< 0.05)

**Fig. 3:**
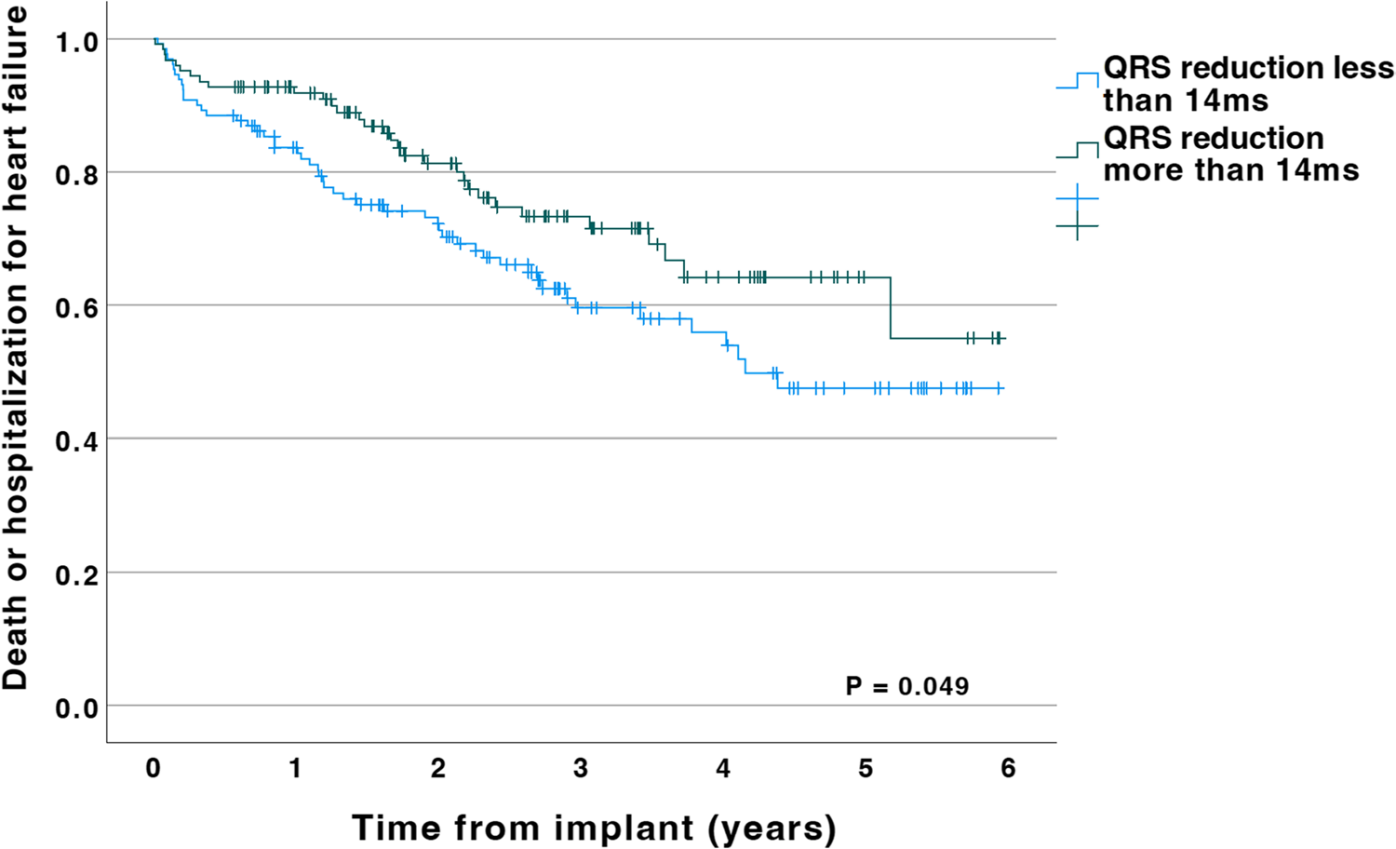
Kaplan-Meier curve showing survival free of heart failure hospitalization stratified for reduction of QRS duration during CRT (cutoff is the median value, a reduction of 14 ms)

**Fig. 4:**
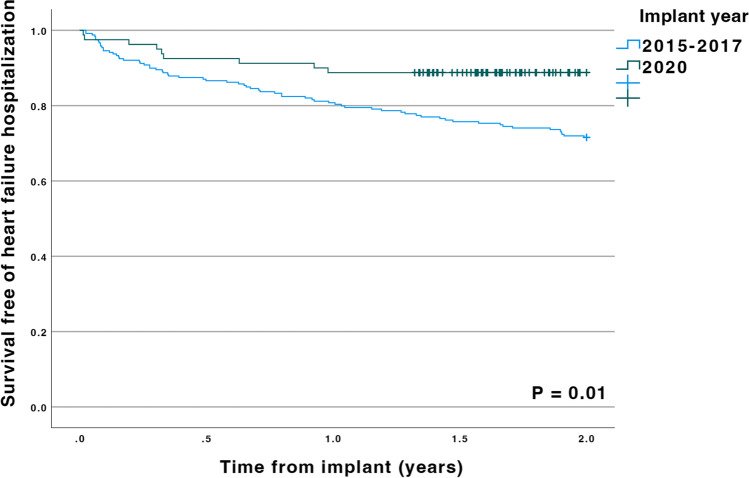
Kaplan Meier curve showing survival free of heart failure hospitalization stratified for implant period (2015–2017 vs. 2020) and truncated to 2 years of follow-up

In Kaplan Meier analysis a QRS reduction > 14 ms was associated with a lower risk of death or heart failure hospitalization (see Fig. 3, *p* = 0.049). When comparing the cohort from 2020 (with the full effect of the optimization procedure, − 24.5ms QRSd reduction on average) with the control cohort, the patients from 2020 had a significantly better survival free of heart failure hospitalization (see Fig. 4, *p* = 0.01).

## Discussion

We show that it is feasible to obtain a larger QRSd reduction in an all-comer CRT-treated population, by using an individualized optimization strategy on top of, or instead of, the device-based optimization algorithms. Despite similar baseline demography and baseline QRSd, the use of a structured optimization resulted in narrower paced QRS complex, and this was in turn associated with a lower risk of heart failure hospitalization and all-cause mortality. Overall, there were only minor differences in the programmed delays between the intervention and control periods, suggesting that there is no general rule to shorten or prolong the intervals in order to achieve larger QRS reduction, but rather that individualization of the AV and VV intervals is key. There was a trend for longer AV delays in the intervention period, which may have allowed for more fusion with intrinsic conduction in the right bundle branch, thereby narrowing the QRS complex and providing better ventricular synchrony.

### Rationale for AV and VV optimization in relation to QRS reduction

There are several pathophysiologic advantages of optimizing the AV interval in CRT. Many patients with LBBB also have a prolonged PR interval and hence there is ineffective LV filling resulting in diastolic mitral regurgitation and fusion of the E and A waves which can be visualized with echocardiography. CRT can overcome this by the programming of shorter AV intervals, but too short an AV delay may result in early closure of the mitral valve prior to actual systole, with the risk of diastolic mitral regurgitation and again ineffective LV filling. Too short AV delays can also be insufficient for optimal filling in the typically large left ventricle of a heart failure patient, which may also have a significant diastolic dysfunction with elevated filling pressures, further compromising LV filling in diastole. Based on this knowledge, the first optimization strategies employed echocardiography, using either a computed “optimal” delay to allow for the best LV filling (Ritter’s method) or an iterative testing-method to determine which setting resulted in the best velocity time integral flow across the aortic or mitral valve (iterative method) [10, 11]. The landmark CRT studies employed various strategies for AV optimization; Care-HF and MIRACLE used echocardiography optimization, COMPANION used a device-based electrical delay algorithm, RAFT CRT used short fixed AV-delays and the MADIT-CRT used no specific AV optimization [1, 12–14]. Device-based algorithms have typically been validated in non-inferiority studies compared to echocardiography-based settings, using LV remodeling as a primary surrogate endpoint [8, 9, 15, 16].

None of the abovementioned validation studies have focused on QRS narrowing as a primary target in CRT, but in a pilot study, Varma et al. recently used the SYNC AV algorithm (Abbott) as a base and then added an individually tailored AV delay “on top of” the device-based suggestion [5]. The SYNC AV algorithm measures the intrinsic AV interval and then subtracts a fixed time (default – 50 ms) to time LV activation for optimal fusion with the intrinsically activated right bundle wavefront. The authors investigated several AV delays and were able to show that the optimal offset varied between − 10 and −60 ms, and that mean QRSd narrowing varied between − 12% (standard BiV pacing with fixed AV delay 140/110 ms) to − 24% (optimal SYNC AV offset). This is in line with the results of our study, where we expand on the previous findings by showing that additional QRS narrowing is feasible, regardless of the device brand and intrinsic algorithm, using a structured approach. LV-only pacing with the aCRT algorithm (Medtronic) has been shown to produce similar improvements in cardiac function compared to biventricular pacing, but a higher proportion of super-responders [17]. In our cohort, the increased use of LV-only pacing algorithms may therefore have provided additional beneficial effects in the intervention group, on top of QRS duration reduction effects. However, in Cox regression analysis, the association with clinical outcome was not significant.

Optimization of VV intervals has not been prospectively evaluated in larger studies, and if it has been evaluated, it has usually been in combination with AV interval optimization, and hence, the effect of additional VV optimization is difficult to tease out [18]. Nevertheless, VV interval optimization is part of all major vendors’ programmable options. The intrinsic algorithms focus on the delta between delays when pacing from the RV electrode and sensing from the LV electrode, and vice versa. Optimizing the VV delay can theoretically be of value, for instance if there is scar surrounding one of the electrodes, making the initial wave-front propagation slower in a unidirectional fashion, manifested by variability in RV-sensing vs. LV-sensing times, and a longer spike-Q interval on the ECG (see central illustration). The clinical impact of optimizing the VV interval remains to be proven, but we hypothesized that if VV optimization can further enhance the QRSd reduction after electrode and AV intervals are optimized, then it may possibly contribute to a better clinical outcome as well.

### QRS duration reduction in relation to clinical outcome in CRT

No prospective randomized trials with clinical outcome as endpoint have investigated a pure QRSd reduction strategy such as ours, and QRSd reduction has not been uniformly reported in the major clinical trials. However, some trials have shown that larger QRSd reduction correlates to better clinical outcome and reverse remodeling [19], and in a recent systematic meta-analysis of 1524 patients from 5 prospective and 6 retrospective studies, there was a significant association between QRSd reduction and favorable echocardiographic response to CRT [20]. In our study the clinical effect was evident only when comparing the year with the best QRSd reduction (i.e., 2020) versus the control years, implying that a substantial relative additional reduction is required for a translation into better clinical outcome compared to device-based algorithms alone. This requires some time and expertise on the part of the nurse or physician who performs the optimization, but after a run-in phase, it should not take more than 20 additional minutes per patient—time well spent if clinical outcomes can be improved.

### Limitations

This was a retrospective single-center study, with the inherent limitations of such a design. The implants were performed during a 6-year period, and the control group had longer follow-up since they were implanted earlier. Even though device-related differences such as activation of LV-only algorithms and LV lead position were not significant in Cox regression models, the combined effect of these differences may have had an interaction with clinical outcome, favoring the intervention group. There may also be residual confounding between the groups, based on changes in referral patterns during this time period, even though baseline demography was similar between the groups, and multivariable adjustment was performed. The association between QRSd reduction and clinical outcome was recorded in the entire cohort, but this does not necessarily mean that the intervention on CRT optimization had a causal effect on the clinical outcome.

## Conclusion

Implementing an ECG-based general strategy of CRT device optimization by aiming for shorter QRS duration is feasible in a structured clinical setting, and results in larger reductions in QRS duration post-implant. In patients with larger QRS reduction, compared to those with smaller QRS reduction, there is an association with a lower risk of mortality and heart failure hospitalization. If confirmed in prospective trials, this strategy may become useful for improving clinical outcome for CRT recipients, regardless of device brand and underlying etiology of heart failure.
